# Genomic Library Screens for Genes Involved in n-Butanol Tolerance in *Escherichia coli*

**DOI:** 10.1371/journal.pone.0017678

**Published:** 2011-03-08

**Authors:** Luis H. Reyes, Maria P. Almario, Katy C. Kao

**Affiliations:** Department of Chemical Engineering, Texas A&M University, College Station, Texas, United States of America; University of Groningen, Netherlands

## Abstract

**Background:**

n-Butanol is a promising emerging biofuel, and recent metabolic engineering efforts have demonstrated the use of several microbial hosts for its production. However, most organisms have very low tolerance to n-butanol (up to 2% (v/v)), limiting the economic viability of this biofuel. The rational engineering of more robust n-butanol production hosts relies upon understanding the mechanisms involved in tolerance. However, the existing knowledge of genes involved in n-butanol tolerance is limited. The goal of this study is therefore to identify *E. coli* genes that are involved in n-butanol tolerance.

**Methodology/Principal Findings:**

Using a genomic library enrichment strategy, we identified approximately 270 genes that were enriched or depleted in n-butanol challenge. The effects of these candidate genes on n-butanol tolerance were experimentally determined using overexpression or deletion libraries. Among the 55 enriched genes tested, 11 were experimentally shown to confer enhanced tolerance to n-butanol when overexpressed compared to the wild-type. Among the 84 depleted genes tested, three conferred increased n-butanol resistance when deleted. The overexpressed genes that conferred the largest increase in n-butanol tolerance were related to iron transport and metabolism, *entC* and *feoA*, which increased the n-butanol tolerance by 32.8±4.0% and 49.1±3.3%, respectively. The deleted gene that resulted in the largest increase in resistance to n-butanol was *astE*, which enhanced n-butanol tolerance by 48.7±6.3%.

**Conclusions/Significance:**

We identified and experimentally verified 14 genes that decreased the inhibitory effect of n-butanol tolerance on *E. coli*. From the data, we were able to expand the current knowledge on the genes involved in n-butanol tolerance; the results suggest that an increased iron transport and metabolism and decreased acid resistance may enhance n-butanol tolerance. The genes and mechanisms identified in this study will be helpful in the rational engineering of more robust biofuel producers.

## Introduction

There has been renewed interest in the four-carbon alcohol, n-butanol, within the scientific and industrial fields due to its potential as an alternative liquid fuel. n-Butanol has physiochemical properties comparable to gasoline, allowing its use as a fuel replacement in internal combustion engines without any modification [1]. Currently, members of the *Clostridia* genus are the only native n-butanol producers known [2], [3]. The solvent production in *Clostridia* is coupled to its complex growth phases, which creates difficulties in the engineering of the organism for improved n-butanol production. The complex growth and production phases and the strict anaerobic nature of the native producers have prompted researchers to pursue heterologous hosts for biobutanol production. In the last few years, with the advances in metabolic engineering, non-native producers of n-butanol such as *Escherichia coli* [4]–[6], *Saccharomyces cerevisiae* [7], *Lactobacillus brevis* [8], *Pseudomonas putida* [9] and *Bacillus subtilis* [9], have been demonstrated as potential hosts for use in n-butanol production. However, n-butanol is highly toxic to microorganisms [10]–[12], with most organisms able to tolerate up to 2% (v/v). An exceptional example corresponds to several adapted *P. putida* strains reported to be able to tolerate concentrations of n-butanol higher than 3% (v/v) in rich medium supplemented with glucose; however the tolerance level of the strains without glucose supplementation or in minimum medium were still 1%–2% (v/v) [13]. Understanding the mechanisms involved in n-butanol response can help to facilitate the engineering of production hosts for improved tolerance.

The toxic effects of n-butanol are believed to result from increased membrane fluidity in the presence of the solvent, disrupting the functions of membrane components [14]. Solvents affect the membrane by disrupting their fatty acid and protein structure. These disruptions alter membrane fluidity [15], impair internal pH regulation [10], disrupt protein-lipid interactions [15] and negatively impact energy generation by inhibiting nutrient transport [10]. Bacteria and other microorganisms can adopt diverse mechanisms to overcome the action of organic solvents. Examples of those mechanisms include: *i.* changes in the hydrophobicity of the outer envelope [16], *ii.* alterations of the cytoplasmic membrane, modifying its structure by changing the saturation of the fatty acids in the phospholipid layer [16], *iii.* changes in the permeability of the membrane by modifications of the lipopolysaccharides and porins [17], [18] of the outer membrane, and *iv.* enhanced efflux pump activity to excrete the solvent present in the cytoplasm [19].

Transcriptional analyses and genomic libraries have been used to investigate the molecular mechanisms involved in n-butanol tolerance in *C. acetobutylicum*. Tomas *et al* [20], using transcriptional analysis, determined that genes involved in general stress response and solvent formation in *C. acetobutylicum*, were upregulated under n-butanol stress. In a study using a *C. acetobutylicum* genomic library enrichment, overexpression of genes encoding for transcriptional regulators, specifically the genes CAC0003 and CAC1869 were identified to increase n-butanol tolerance by 13% and 81% respectively [15]. The response of *E. coli* to isobutanol via transcriptional analysis has elucidated that quinone malfunction and the action of ArcA are some of the key perturbations during solvent stress [21]. Rutherford *et al* [22] showed that n-butanol stress response in *E. coli* share components with other common stress responses. These commonalities include changes in respiratory functions (*nuo* and *cyo* operons), responses to heat shock, oxidative, and cell envelope stress (*rpoE*, *clpB*, *htpG*, *cpxR*, *cpxP*, *sodA*, *sodC*, and *yqhD*), and changes in metabolite transport and biosynthesis (*malE* and *opp* operon). These studies demonstrated that the response to n-butanol is a complex phenotype, involving multiple mechanisms.

Thus far, few genes have been directly identified to be involved in enhanced tolerance to n-butanol. Using an *E. coli* genomic library enrichment strategy, we identified several candidate genes that are involved in n-butanol tolerance. Candidate genes that are enriched or depleted from the genomic enrichment were tested using overexpression and knockout libraries, respectively. Several of the candidate genes tested were confirmed to reduce the growth inhibitory effects of n-butanol on *E. coli*.

## Results and Discussion

### Genomic library construction and description of n-butanol challenge

An *E. coli* genomic library with an approximately seven-fold-coverage of the *E. coli* genome was generated (details are described in the *Materials and Methods* section). The genomic library was exposed to increasing concentrations of n-butanol (0.5%, 0.9%, 1.3%, and 1.7% (v/v)) via batch serial transfers. To reduce false positives, control enrichments in the absence of n-butanol were included. Samples were collected after each step in the n-butanol challenge for subsequent analysis to identify the genes that are enriched or depleted in the presence of n-butanol.

### Identifying enriched genes via array-CGH

**Figure 1:**
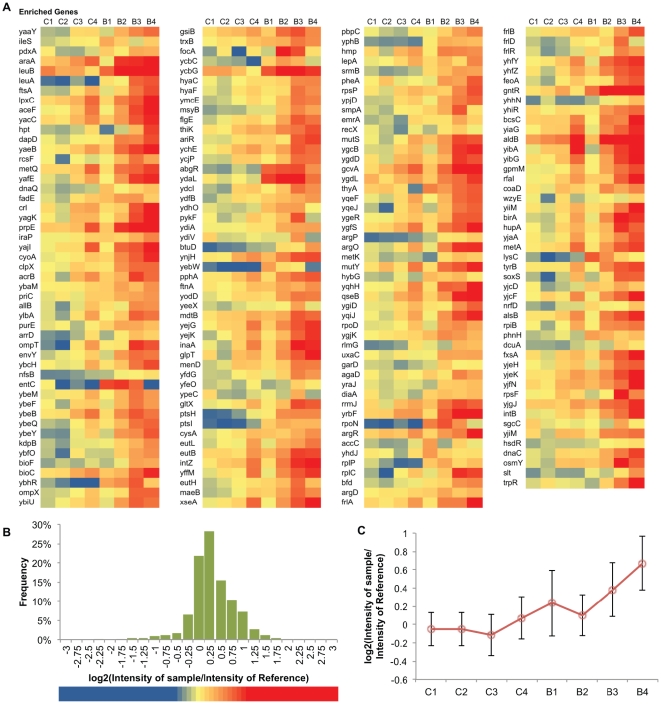
Profiles of genes significantly enriched in the n-butanol challenge. **A**. Heat map of all genes enriched. **B.** Histogram of the range of normalized *log_2_(Intensity of sample/Intensity of reference)*. The colored bar at the bottom part of the figure is the legend for Figure 3A. **C.** The averaged profile.

The plasmids from the genomic library after each step of the serial n-butanol challenge were extracted and hybridized to Comparative Genome Hybridization microarrays (array-CGH), using the unchallenged (original) *E. coli* genomic library as reference. The data obtained from the array-CGH were analyzed as described in the *Materials and Methods* section. Some of the enriched genes identified from the n-butanol challenge may indeed confer enhancements in n-butanol tolerance. However, certain genes may be enriched as a result of metabolic enhancement (*e.g.* more efficient nutrient uptake and utilization) rather than solvent tolerance. Since, the enriched genes from the controls likely confer general growth advantage through metabolic enhancements, any gene enriched in the n-butanol-challenged libraries that was also enriched in the control experiments was removed from further analysis. In the end, a total of 193 candidate genes were identified to be enriched from the n-butanol challenge. Their enrichment profiles are shown in **Figure 1**.

Among the enriched set of genes shown in **Figure 1**, approximately 30% have membrane-related functions based on Gene Ontology (GO) terms (whereas around 17% of the currently annotated *E. coli* genes are membrane-related), which corresponds with the main cellular response to the presence of other organic solvents [13], [23]–[25]. The main groups of enriched membrane-related genes are those constituting efflux pumps and anti-porters, amino acid and sugar transporter systems, membrane lipoproteins, multidrug resistance and stress response genes. **Table 1** shows the list of enriched genes with membrane-related functions.

**Table 1:** Membrane related genes enriched in the n-butanol challenge.

Function	Genes enriched
Efflux pump and anti-porters	*acrB, argO, emrA, focA* and *ybhR*
Amino acid and sugar transporter systems	*agaD, alsB, btuD, dcuA, frlA, glpT, gsiB, kdpB, metQ, sgcC, ycjP* and *yjeH*
Membrane lipoproteins	*cyoA, eutH, eutL, hyaC, ompT, ompX, rfaI, smpA, yajI, yfdG, ygdD, yjcD* and *ypjD*
Multidrug resistance	*acrB, emrA, mdtB* and *ychE*
Stress response	*ompT, yjaA* and *yodD*

The genes *acrB, argO, mdtB, emrA* were enriched in the n-butanol challenge. Studies in *E. coli* have shown that the AcrAB efflux system is important in multidrug, cyclohexane, n-hexane and n-pentane resistance [26]. Our result suggests that AcrB plays a role in n-butanol tolerance as well, possibly by alleviating the cytoplasmic concentration of solvent. Similar conclusions can be drawn for the arginine effluxer (ArgO), the MdtABC multidrug export system [27] and the EmrAB transport system [28]. Enrichment of genes involved in amino acid and sugar transport, such as *argD, argR, dapD, lysC, leuA* and *leuB*, suggest that higher energy requirements may be needed to overcome the solvent challenge. The enrichment of genes such as *ompX*, which is a part of a complex regulatory network involved in the control of outer membrane adaptability and permeability [29], and *smpA*, encoding for the small outer-membrane lipoprotein regulated by σ^E^ [30], potentially suggest that one mechanism for n-butanol resistance is by preventing n-butanol influx to the cytosol and the disruption of the cell envelope. The xanthine/uracil permease (YjcD), enriched in our experiment, has been predicted to belong to the *purR* regulon [31], which has been identified to be involved in organic solvent tolerance [32]. YjaA and YodD are proteins involved in stress response of *E. coli* to hydrogen peroxide, cadmium and acid [33], and our data suggests a potential link of those genes with tolerance to n-butanol. SoxS, a transcriptional activator, has been found as an important transcription factor in the nitric acid, hydrogen peroxide and oxidative stress [34], [35], and tolerance to multiple drugs [36] and cyclohexane [26], possibly via lipopolysaccharide modification.

A gene ontology analysis of the enriched set of genes, using the toolkit GOEAST (Gene Ontology Enrichment Analysis Software Toolkit) [37], was carried out to identify significantly enriched Gene Ontology (GO) groups in our dataset. The enriched GO terms from the list of enriched genes are summarized in **Table 2**.

**Table 2:** Gene Ontology terms enriched in the enriched set of genes.

GO ID	Term	Log odd-ratio	Corrected p-value
GO:0003700	Sequence-specific DNA binding transcription factor activity	0.62	0.07
GO:0016564	Transcription repressor activity	0.90	0.07
GO:0050897	Cobalt ion binding	1.71	0.06
GO:0030145	Manganese ion binding	1.03	0.09
GO:0006525	Arginine metabolic process	1.71	0.06
GO:0009085	Lysine biosynthetic process	2.64	0.01
GO:0019867	Outer membrane	0.97	0.07
GO:0009102	Biotin biosynthetic process	2.93	0.01
GO:0030955	Potassium ion binding	2.20	0.02
GO:0046912	Transferase activity, transferring acyl groups, acyl groups converted into alkyl on transfer	2.93	0.02
GO:0009098	Leucine biosynthetic process	3.20	0.02
GO:0006352	Transcription initiation	2.93	0.02
GO:0016987	Sigma factor activity	2.71	0.03
GO:0044011	Single-species biofilm formation on inanimate substrate	3.52	0.02
GO:0070301	Cellular response to hydrogen peroxide	3.10	0.02

Biotin (*birA*, *bioC*, and *bioF*) and amino acid biosynthesis (arginine, lysine, and leucine) were among the functions enriched from the GO-term analysis. Enzymes requiring biotin include acetyl-CoA carboxylase, pyruvate carboxylase, propionyl-CoA carboxylase, methylcrotonyl-CoA carboxylase, geranoyl-CoA carboxylase, oxaloacetate decarboxylase, methylmalonyl-CoA decarboxylase, transcarboxylase and urea amidolyase, which are involved in a variety of different processes such as fatty acid biosynthesis, amino acid metabolism and the citric acid cycle. In fatty acid biosynthesis, biotin has been demonstrated to affect the lipid composition of the cell wall and membrane of *E. coli* [38]; cells deficient in biotin showed a decrease in unsaturated fatty acids, the presence of unsaponifiable lipid material and the lack of a lipopolysaccharide fraction in the cell wall and membrane [38]. One of the microbial defense mechanisms against organic solvents involves alterations of the cytoplasmic membrane structure, either by modifying the degree of saturation of the fatty acids, isomerization of unsaturated fatty acids, or altering the dynamics of the phospholipid turnover, thereby reestablishing the fluidity and stability of the membrane [16]. Modifications of the lipopolysaccharides in the presence of organic solvents has also been identified [17]. Thus, the enrichment in biotin biosynthesis genes suggests that increased biosynthesis of biotin may help to enhance cell wall and/or membrane integrity. However, the enrichment of *birA*, which is a repressor of the biotin biosynthesis genes, runs counter to this argument. Since BirA also serves the role of the biotin-ligase in the activation of the enzyme acetyl-CoA carboxylase (ACC) [39], which is the first committed step in fatty acid biosynthesis, the enrichment of *birA* seems to suggest that the activation of ACC may have a larger effect on n-butanol tolerance than reduction in biotin biosynthesis. Several ion-binding proteins were enriched in our studies (*allB, metK, pdxA, araA, leuB, menD, pphA and pykF*). Enrichment in the potassium transporter, *kdpB*, suggests that ion transport may be involved in n-butanol tolerance, possibly by increasing the motive force of many efflux pumps systems [40]. In addition, several genes with transcriptional regulation-related functions, such as *srmB*, *rpoD*, *rpoN*, *rplP*, *rplC*, *rpiB* and *rpsF*, were also enriched. Borden and Papoutsakis also found that 4 out of 16 loci that were enriched in a *C. acetobutylicum* genomic library under n-butanol stress were transcriptional regulators [15]. This suggests that global transcriptional perturbations may be involved in n-butanol tolerance.

### Analysis of genes enriched during n-butanol challenge through the use of an overexpression library

To validate whether the genes enriched from the n-butanol-challenged libraries were indeed involved in enhanced n-butanol tolerance, we used clones from the ASKA collection [41], which is an ORFeome library collection for *E. coli* K-12. Two parameters were calculated to determine the enhancement in n-butanol tolerance due to overexpression of a gene, the Improvement in the Inhibitory Effect (IIE) and the Reduction of Specific Growth Rate in absence of n-butanol (RSGR), as described in the *Materials and Methods* section. IIE measure the increase (in percentage) in the n-butanol tolerance (defined as the improvement of the specific growth rate in presence of n-butanol in comparison with the specific growth rate in absence of the solvent) of the overexpression strain in comparison with the wild-type strain. Positive values of IIE signify improvements in n-butanol tolerance in the overexpression strain compared to the wild-type. RSGR measures the change of the specific growth rate due to the overexpression of the gene. Under the hypothesis that an increase in the maximum specific growth rate (µ_max_) is an indication of enhanced tolerance to the solvent, we calculated the parameters IIE and RSGR for each of the strains overexpressing the candidate genes tested. Another alternative measurement to determine the enhancement in n-butanol tolerance is the growth yield. However, based on our data, the specific growth rate seems to be a more sensitive measurement of such improvement (overexpression of some genes decrease the specific growth rate without a significant effect on the growth yield).

We screened 55 out of the 194 genes that were enriched in the n-butanol-challenged library, and identified 11 genes that conferred significant increase in n-butanol tolerance when overexpressed (**Table 3**). Two genes involved in iron metabolism (*entC* and *feoA*) were found to confer a significant increase in n-butanol resistance. Iron metabolism has not been previously associated with enhanced n-butanol tolerance. However, several genes related to iron metabolism were downregulated in *E. coli* under isobutanol stress [21], suggesting a disruption in iron metabolism. Thus, the enhanced n-butanol resistance in *entC* and *feoA* overexpressing strains may be due to the compensatory effects of such a disruption in n-butanol stress. Interestingly, three of the 11 genes (*yibA*, *metA* and *ymcE* [42]–[44]) are heat shock related genes. These genes are under the control of σ^32^, which is a sigma factor that is active under several stress conditions. Overexpression of the outer membrane protease, OmpT, which is active under extreme denaturing conditions [45], was found to increase n-butanol tolerance. The formate transporter, encoded by the gene *focA*, which can also act as an efflux pump that regulates the intracellular formate pool [46], also enhanced n-butanol tolerance when overexpressed.

**Table 3:** Genes that significantly increase n-butanol tolerance when they are overexpressed using ASKA collection.

Clone	IIE	RSGR	p-Value
*ompT*	10.8±0.9%	−13.3±0.7%	0.01
*entC*	32.8±4.0%	−0.8±0.1%	0.05
*yibA*	12.7±0.8%	−8.4±0.3%	0.02
*metA*	14.9±0.9%	−7.2±0.2%	0.01
*alsB*	13.9±1.0%	−12.2±0.6%	0.02
*phnH*	42.4±3.0%	18.4±0.4%	0.01
*feoA*	49.1±3.3%	3.6±0.1%	0.00
*focA*	4.3±0.2%	−15.4±0.3%	0.02
*hyaF*	20.8±2.1%	15.4±0.6%	0.03
*ymcE*	13.2±0.4%	−11.1±0.2%	0.02
*yfdG*	20.3±1.4%	4.9±0.2%	0.00

### Depleted genes

**Figure 2:**
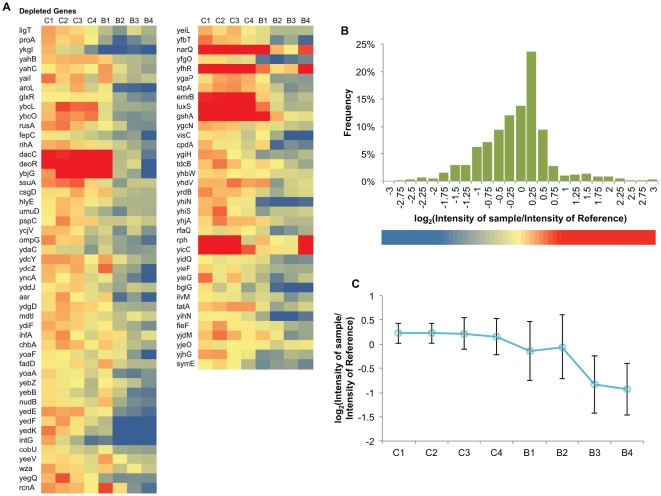
Profiles of genes significantly depleted in the n-butanol challenge. **A**. Heat map of all genes depleted. **B.** Histogram of the range of normalized *log_2_(Intensity of sample/Intensity of Reference*. The colored bar at the bottom part of the figure is the legend for Figure 4A. **C.** The averaged profile.

Along with the enriched genes, depleted genes from the n-butanol-challenged libraries identified in the array-CGH were also analyzed, as some of these genes may help to enhance n-butanol tolerance when their expression is decreased. Similar selection criteria as those used for the enriched gene set were applied to identify and analyze the genes that are significantly depleted. A total of 84 significantly depleted genes were identified (see **Figure 2** for the list of genes).

Analysis of the depleted genes may reveal the possible negative effects of higher expression of these genes under n-butanol stress. Those effects can be grouped in two main categories. The first group are genes that when overexpressed possibly increase the metabolic burden to the cell. Genes like *purP*, which is involved in energized high-affinity adenine uptake [47], [48], and *luxS*, which synthesizes the quorum sensing molecule autoinducer-2 (AI-2) [49], are likely not directly involved in increase n-butanol susceptibility. Their depletion from the library is likely due to the increased metabolic burden. The second group constitutes genes that may increase the concentration of n-butanol in the cell. OmpG, which is a nonspecific and efficient channel for sugar and large solutes [50], may also allow the diffusion of n-butanol into the cell. **Table 4** shows the results of the gene ontology analysis of the set of depleted genes.

**Table 4:** Gene Ontology terms enriched in the depleted gene set.

GO ID	Term	Log odd-ratio	Corrected p-value
GO:0006508	Proteolysis	2.54	0.00
GO:0008360	Regulation of cell shape	1.94	0.09
GO:0008658	Penicillin binding	3.42	0.09
GO:0008236	Serine-type peptidase activity	3.57	0.00
GO:0009081	Branched chain family amino acid metabolic process	2.42	0.05
GO:0009405	Pathogenesis	3.42	0.10
GO:0003984	Acetolactate synthase activity	3.42	0.10
GO:0046654	Tetrahydrofolate biosynthetic process	3.42	0.04
GO:0046930	Pore complex	2.94	0.02
GO:0043190	ATP-binding cassette (ABC) transporter complex	1.89	0.09
GO:0009432	SOS response	2.42	0.05
GO:0015774	Polysaccharide transport	2.57	0.09

### Analysis of genes depleted during the n-butanol challenge using the *E. coli* knockout collection

Strains from the Keio knockout collection [51], [52] were used to examine if deletion of the depleted genes could increase the n-butanol tolerance of *E. coli*. The IIE and RSGR parameters were calculated from the wild-type strain and the deletion mutant in M9 minimal medium at 0% and 0.5% (v/v) n-butanol.

**Figure 3:**
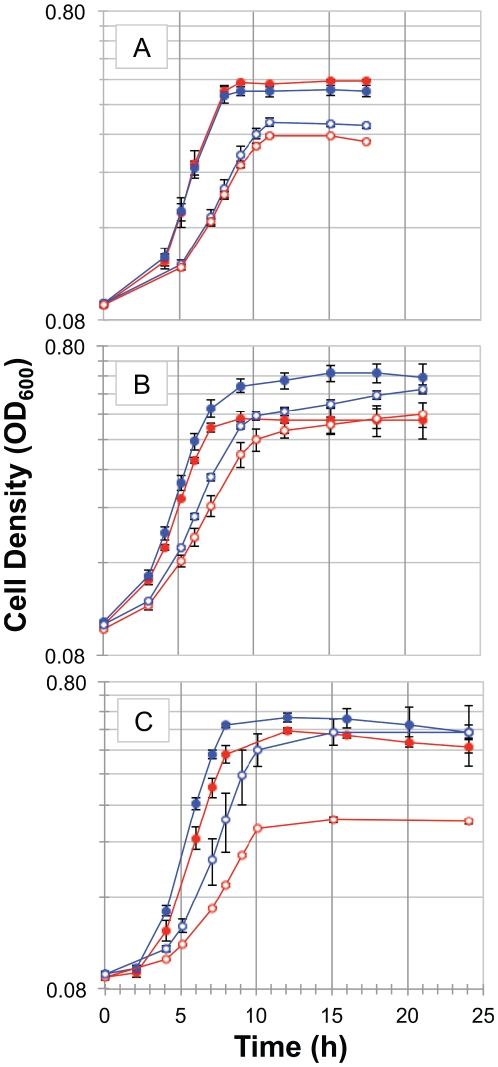
The growth kinetics of A. *ΔygiH*, B. *ΔastE*, and C. *Δrph* vs. wild type. Red lines represent the growth kinetics of wild type in absence (open circle) and presence (solid circles) of 0.5% (v/v) n-butanol. Blue lines represent the growth curves of the deletion strains in absence (open circle) and presence (solid circle) of 0.5% (v/v) n-butanol.

Out of 84 genes tested, three genes were found to significantly reduce the inhibitory effect of n-butanol when they were deleted: *astE, ygiH* and *rph*. The calculated parameters are shown in **Table 5**. Improvements in the relative specific growth rates were observed in all three deletion strains in the presence of n-butanol compared with the wild-type (see **Figure 3**). AstE hydrolyzes N^2^-succinylglutamate into succinate and L-glutamate. L-glutamate has been identified to be involved in acid stress response in *E. coli* [53], [54]. Recent studies have demonstrated that n-butanol response in *Lactobacillus brevis* [55] downregulated the acid stress response significantly (Winkler and Kao, manuscript submitted). Thus, deletion of *astE* may lead to decreased L-glutamate pool, resulting in increased n-butanol tolerance. Deletion of *ygiH*, the gene encoding an inner membrane protein, increased resistance to n-butanol by 14.8±1.2%. Studies have found that PlsY proteins in *Bacillus subtilis* and *Streptococcus pneumoniae* exhibit similarities with YgiH, as they both function as the glycerol-3-phosphate acyltransferases for phospholipid biosynthesis [56]. However, in *E. coli*, the function of PlsY is replaced by PlsB, and PlsX and YgiH play important roles in regulating the intracellular levels of acyl-ACP, an important precursor in the fatty acid biosynthesis. Studies demonstrated that single deletions of the PlsX or YgiH do not strongly affect cell growth, however double deletion is synthetically lethal [56]. The depletion of YgiH suggests that phospholipid biosynthesis [56] may be optimized to the requirements needed to overcome the solvent stress. Deletion of the RNase PH gene, Rph, resulted in an increase in n-butanol tolerance by 48.4±4.1%. However, the *E. coli* strain BW25113, used in this study, has a *rph-* background, with a frameshift mutation inactivating *rph* function. Complete deletion of this gene may ameliorate transcriptional polarity on the *pyrE* gene, increasing pyrimidine biosynthesis [57]. Thus, *rph* most likely is not directly involved in n-butanol tolerance in *E. coli*.

**Table 5:** Genes that significantly enhance n-butanol tolerance when deleted from the *E. coli* genome.

Mutant	IIE	RSGR	p-value
*astE*	48.7±6.3%	−3.3±0.3%	0.00
*ygiH*	14.8±1.2%	12.3±0.6%	0.02
*rph*	48.4±4.1%	−10.2±0.6%	0.01

### Conclusions

Using a genomic library enrichment strategy, we identified genes involved in n-butanol tolerance in *E. coli*. We identified two groups of genes from the n-butanol challenge: genes that were enriched and depleted during the exposure to n-butanol. From the data, we were able to expand the current knowledge on the genes involved in n-butanol tolerance; we observed enrichment of genes involved in membrane functions, transport systems (encoded by *acrB, argO, mdtB* and *emrA*), amino acid transport, sugar transport and stress response proteins. We also found enrichment in genes involved in biotin synthesis (*bioC* and *bioF*), indicating that an increase in this cofactor may help to enhance membrane integrity. Among the depleted genes, we identified genes that when overexpressed may cause undesirable increase in n-butanol inside the cell. We experimentally verified 14 genes that decreased the growth-inhibitory effects of n-butanol on *E. coli*. The overexpression of the iron transport and metabolism related genes, *entC* and *feoA*, increased n-butanol tolerance by 32.8±4.0% and 49.1±3.3%, respectively. Deletion of *astE*, which may lead to decreased L-glutamate (potentially decreasing acid resistance), enhanced n-butanol tolerance by 48.7±6.3%. The genes and mechanisms identified in this study will be useful in the rational engineering of more robust biofuel producers. In addition, since organic solvent tolerance is known as a complex phenotype, there may be potential synergistic effects between different combinations of deletions and overexpressions of genes identified in this work; we will be investigating such effects in subsequent works.

## Materials and Methods

### Bacterial strains, plasmid constructs and genomic library construction

The *E. coli* K-12 strain, BW25113 (Δ(*araD*-*araB*)*567*, Δ*lacZ4787*(::*rrnB*-3), *lambda*-, *rph*-1, Δ(*rhaD*-*rhaB*)568, *hsdR514*), was used in this study. Overnight cultures from frozen stocks were grown in 5 ml of Luria-Bertani broth [58] or on solid LB agar plates supplemented with kanamycin (30 µg/ml) and incubated at 37°C.

Genomic DNA was extracted using DNeasy Blood & Tissue Kit (QIAGEN). The genomic DNA was fragmented to pieces between 2000 and 3000 base pairs using sonication (Ultrasonic Liquid Processor S-4000, Misonix, Inc). The ends of the fragmented DNA were repaired using T4 DNA polymerase (New England Biolabs). The library of repaired DNA fragments were ligated to the pSMART-LC Kan vector (Lucigen Corporation), following the manufacture's instructions and transformed into *E. coli* by electroporation using the Gene PulserMXcell Electroporation System (Bio-rad). Cells (approximately 14,000 colonies) were recovered from the plates and frozen stocks of the genomic library were made and saved at −80°C.

### n-Butanol challenge

The genomic library was inoculated in 25 ml of LB and incubated at 37°C until OD_600_ of approximately 0.6 was reached. A sample was collected to be used as the reference. The enrichment strategy involves the serial transfers of batch cultures in increasing n-butanol concentrations (0%, 0.9%, 1.3% and 1.7% n-butanol v/v) along with the respective controls (enrichment scheme shown in **Figure S1**). For each serial transfer, when the cultures reached the desired OD_600_ (approximately 0.7), a sample was taken, and the plasmids from the enriched libraries were recovered using alkaline lysis procedure [59]. The constructs were verified via PCR, using the primers SL1 5′-CAG TCC AGT TAC GCT GGA GTC-3′ and SR2 5′-GGT CAG GTA TGA TTT AAA TGG TCA GT-3′.

### Comparative genome hybridization microarray (array-CGH)

The plasmid DNA (5 µg) isolated from each step of the enrichment, was digested at 37°C for two hours with 10 units each of AluI and RsaI (Invitrogen Corporation) in a reaction containing 10 mM MgCl_2_ and 50 mMTris-HCl (pH = 8.0). Samples were cleaned using Zymo Clean & Concentrate-5 columns (Zymo Research), and eluted in TE (pH = 8.0). The fragmented plasmid DNA was labeled and hybridized using the BioPrime® Total kit (Invitrogen Corporation) for Agilent aCGH, following manufacture's protocols.

Each labeled sample along with the differentially labeled reference were hybridized to Agilent *E. coli* catalog arrays (*E. coli* gene expression microarray, Agilent Technologies) according to the manufacture's instructions. The arrays were scanned using the GenePix 4100A Microarray Scanner and image analysis performed using GenePix Pro 6.0 Software (Molecular Devices). The Microarray Data Analysis System software was used to normalize the data using LOWESS based normalization algorithm [60], [61]. Subsequently, a Student's t-test was used to identify the genes that are statistically significantly enriched or depleted (p-value below 5%) in the n-butanol challenge. The selected genes were clustered via Cluster Affinity Search Technique [62], using the software MeV (Multiexperiment viewer) from the TM4 Microarray Software Suite [63], to group genes with similar enrichment profiles.

### Growth kinetic parameters calculated for the genes enriched (via ASKA collection) and depleted (via Keio Collection)

The parameters “Improvement in the Inhibitory Effect” (IIE) and “Reduction of Specific Growth Rate in absence of n-butanol” (RSGR) were calculated using Equations 1 and 2 respectively. Those parameters were determined by measuring the maximum specific growth rate (µ_max_) of the wild-type and the clone (carrying the overexpression plasmid or the deletion clone) in M9 minimal medium (supplied with 5 g/L glucose) at two different concentrations of n-butanol, 0% and 0.5% (v/v). The growth kinetics for each strain was measured using a TECAN Infinite M200 Microplate reader (TECAN). Four biological replicas were obtained per sample. A Student's t-test was carried out on the four biological replicates to determine if there was a significant improvement in the n-butanol tolerance when the gene was overexpressed or deleted from the genome.
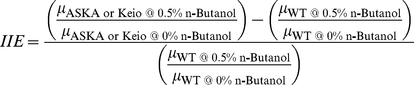
(1)

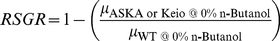
(2)


Where µ_ASKA or Keio @ 0.5% n-Butanol_ and µ_WT @ 0.5% n-Butanol_ are the specific growth rates of the overexpression/deletion strain or wild-type strain in 0.5% (v/v) n-butanol, respectively, and µ_ASKA or Keio @ 0% n-Butanol_ and µ_WT @ 0% n-Butanol_ are the specific growth rates of the overexpression/deletion strain or wild-type strain in the absence of n-butanol, respectively.

### Data Availability

All raw data is MIAME compliant and have been deposited in the GEO database with accession number GSE26223.

## Supporting Information

Figure S1 **n-Butanol challenge strategy.** The library was serially transferred in batch cultures with increasing n-butanol concentration. Control serial transfers in the absence of n-butanol was included.
(EPS)
